# Supplementary material for: Nicotinamide Counteracts the Detrimental Effect of Endothelin-1 on Uterine Decidualization During Early Pregnancy by Influencing EDNRB
Source: Cells. 2025 Oct 22;14(21):1645. doi: 10.3390/cells14211645 (PMC12608920; doi:10.3390/cells14211645)
Supplement: Supplementary file 1 [file cells-14-01645-s001.zip › cells-3926256-supplementary.pdf]

Supplemental Information

Nicotinamide counteracts endothelin-1's detrimental effect on uterine decidualization during early pregnancy by influencing EDNRB

Yuye Wang [1], Qing Ma [1], Meitong Chen[1], Yukako Kayashima [1], Jiayi Zhou [2] , Balaji Rao [3], Jessica L Bowser [1], Xianwen Yi[1], Nobuyo Maeda-Smithies [1] and Feng Li [1*]

[1] Department of Pathology and Laboratory Medicine, The University of North Carolina, Chapel Hill, NC 27599, USA.

[2] Department of Nutrition, Gillings School of Global Public Health, University of North Carolina at Chapel Hill, Chapel Hill, NC 27599, USA

[3] Department of Chemical and Biomolecular Engineering, Golden LEAF Biomanufacturing Training and Education Center, North Carolina State University, Raleigh, North Carolina, USA

*Correspondence to: Feng Li Ph.D., Department of Pathology and Laboratory Medicine, The University of North Carolina, Chapel Hill, NC 27599, USA.

Phone: 919-597-0864. Fax: 919-966-8800.

E-mail: lif@med.unc.edu

Table S1. Primers and probes for qRT-PCR

Gene	Type	Sequence (5'-3')
m-Edn1	Forward	CAG CAG TTA GTG AGA GGA AG
	Reverse	GAC GCT GTT TCT CAT GGT CT
	Probe	FMA-TC CCG AGC GCG TCG TAC CGT ATG- TAMRA
h-EDN1	Forward	TGC CAC CTG GAC ATC ATC TG
	Reverse	ACG CTT GGA CCT GGA AGA AC
	Probe	FMA-AC CTG GTT TGT CTT AGG TGT TCC TC- TAMRA
m-Ednra	Forward	TCT CTG CAA GCT GTT CCC CT
	Reverse	AGC CAC TGC TCT GTA CCT GT
	Probe	FAM-CC TGC AGA AGT CCT CCG TGG GC- TAMRA
m-Ednrb	Forward	TGG CCA TTT GGA GCT GAG AT
	Reverse	CAG CTC GAT ATC TGT CAA TAC
	Probe	FAM-TG TAA GCT GGT GCC CTT CAT ACA GAA GGC- TAMRA
h-EDNRB	Hs00240747_m1	
h-GAPDH	Forward	GAA GGT GAA GGT CGG AGT C
	Reverse	GAA GAT GGT GAT GGG ATT TC
	Probe	FAM-CA AGC TTC CCG TTC TCA GCC- TAMRA
m-Hif1	Forward	CAT CTG ACC AAA ACT CAC CAT G
	Reverse	GAC ATA TCC ACC TCT TTT GGC
	Probe	FMA-AG GAC AAG TCA CCA CAG GAC AGT ACA G- TAMRA
h-IGFBP	Hs00236877_m1	
h-PRL	Hs00168730_m1	
m-Vegf	Forward	CGG TTT AAA TCC TGG AGC GT
	Reverse	ACG TCT GCG GAT CTT GGA CA
	Probe	FAM-CT GTG AGC CTT GTT CAG AGC GGA G- TAMRA
h-VEGF	Hs00900055_m1	
h-WNT4	Hs01573505_m1	
18s	Forward	AGA AAC GGC TAC CAC ATC CA
	Reverse	CTC GAA AGA GTC CTG TAT TGT
	Probe	FAM-AG G CAG CAG GCG CGC AAA TTA C—TAMRA

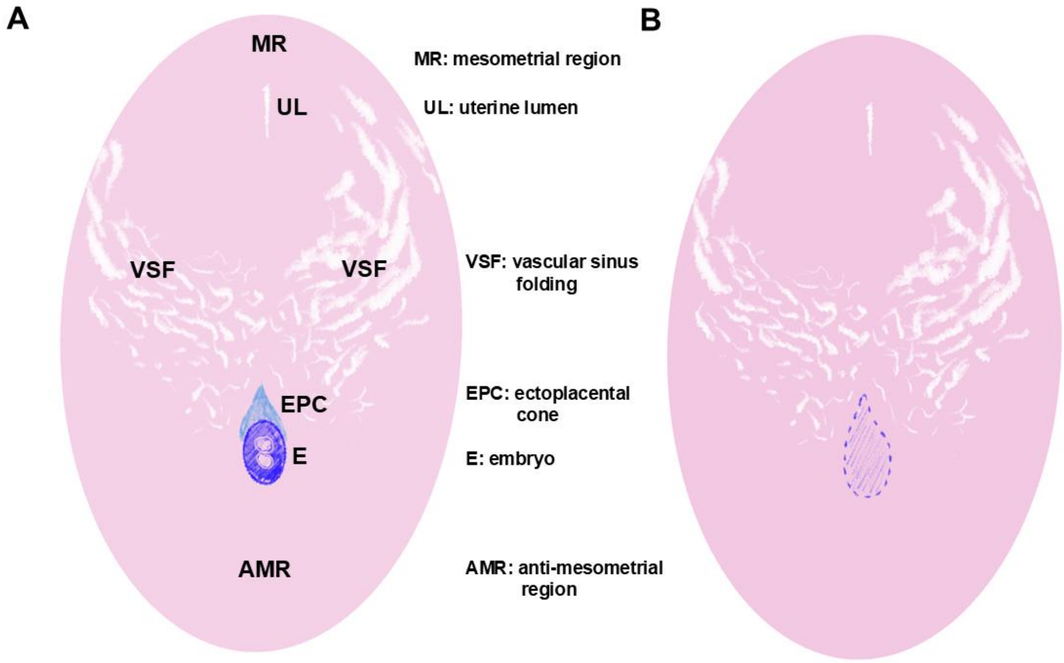

Supplemental Figure S1. A. Illustration of the structures of implantation sites at 7.5 days post coitus. **B.** Illustration of the maternal decidual region (excluding broken line enclosed embryo and ectoplacental cone) was used to determine mRNA and protein for the current research.

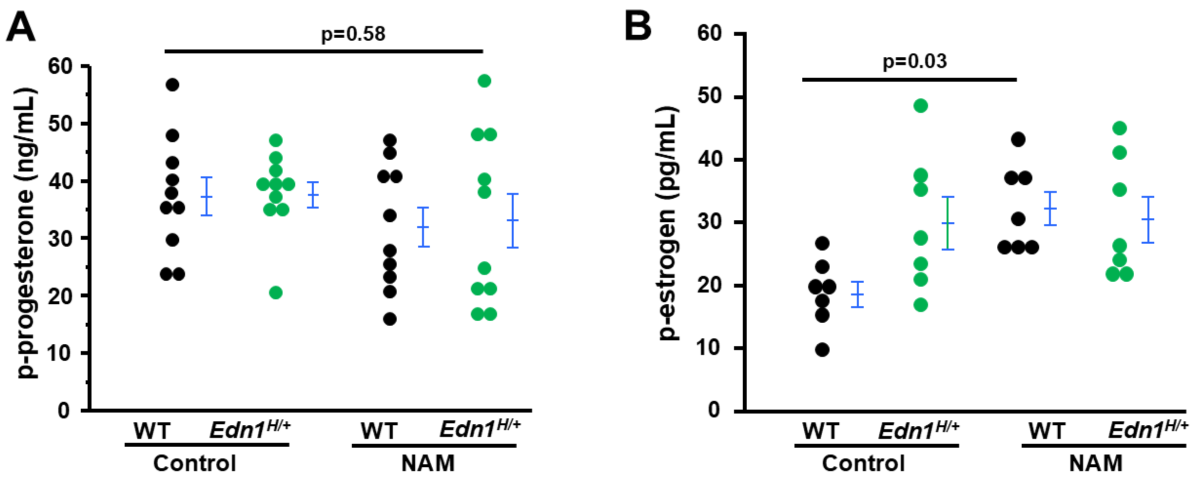

Supplemental Figure S2. Plasma progesterone (A) and estrogen (B) levels in four groups of mice. A. Plasma (p) progesterone. n=10. ANOVA analysis F=0.58, Degree of freedom: group=3, Error=36, C. Total=39. **B**. Plasma (p) estrogen concentration. n=7. Tukey-Kramer HSD.

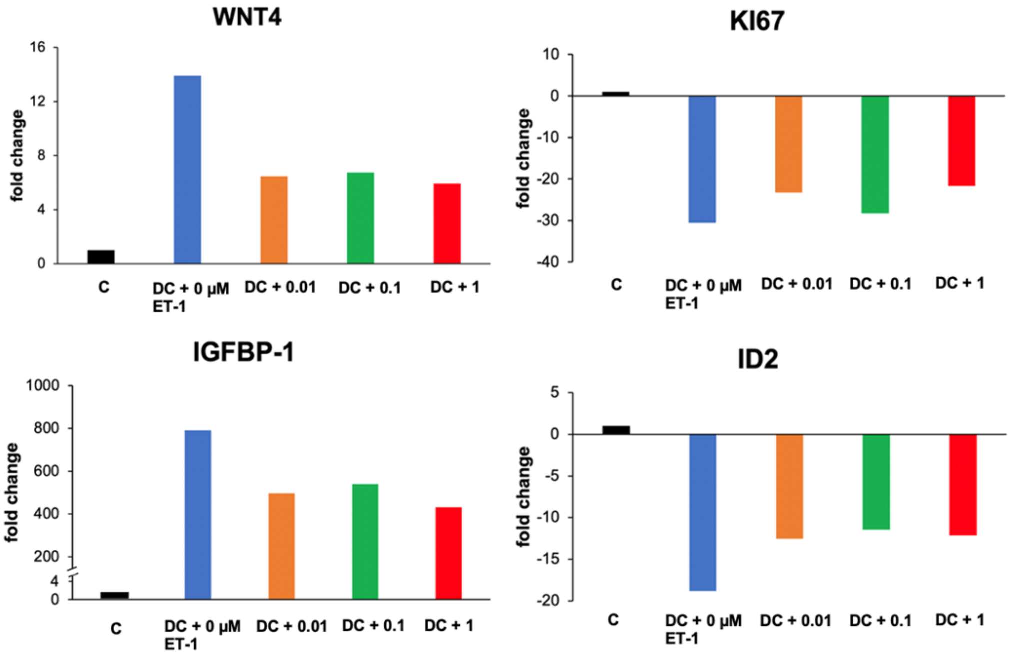

Supplemental Figure S3. The effects of ET-1 on the expression of markers of decidualization and proliferation in human endometrial stromal cells. Three days after being treated with a decidualization cocktail (DC), cells were subjected to RNA-seq analysis. The upregulated expression of markers of decidualization (WNT4 and IGFBP-1) in DC cells was significantly decreased by ET-1, and the three doses had similar effects. The downregulated expression of markers of proliferation (KI67 and ID2) during differentiation (decidualization) was increased by ET-1, and the three doses had similar effects again. C: control cells without addition of DC and ET-1. Each group had one biological sample.

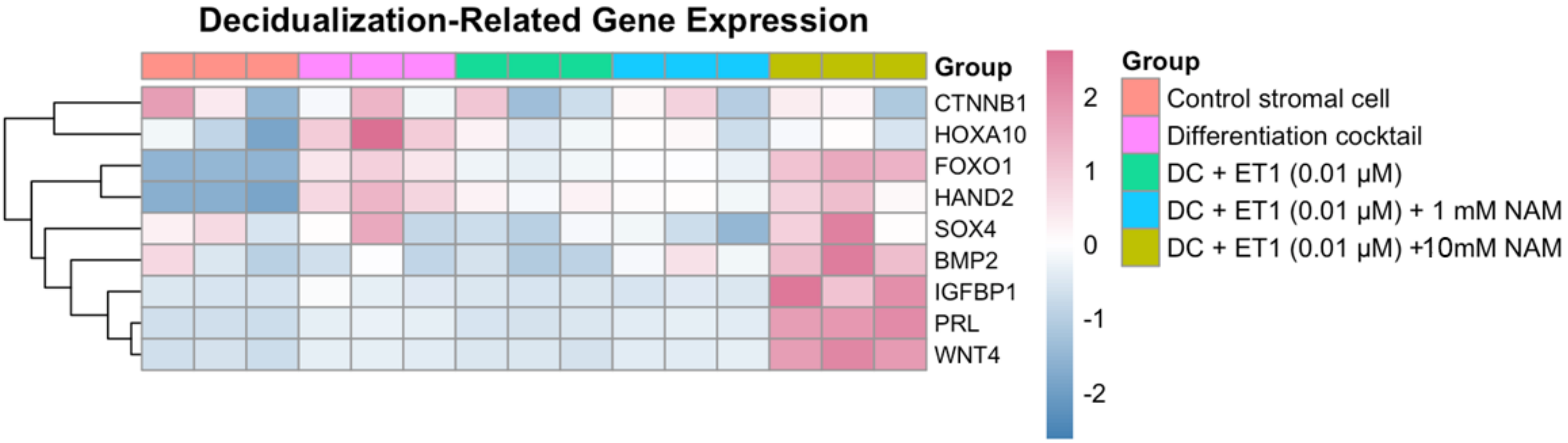

Supplemental Figure S4. Heatmap of genes related to decidualization. Three days after being treated with a decidualization cocktail (DC), cells were subjected to RNA-seq analysis. The upregulated expression genes in DC cells were significantly decreased by ET-1, and NAM (10 mM) completely restored the expression of these genes.

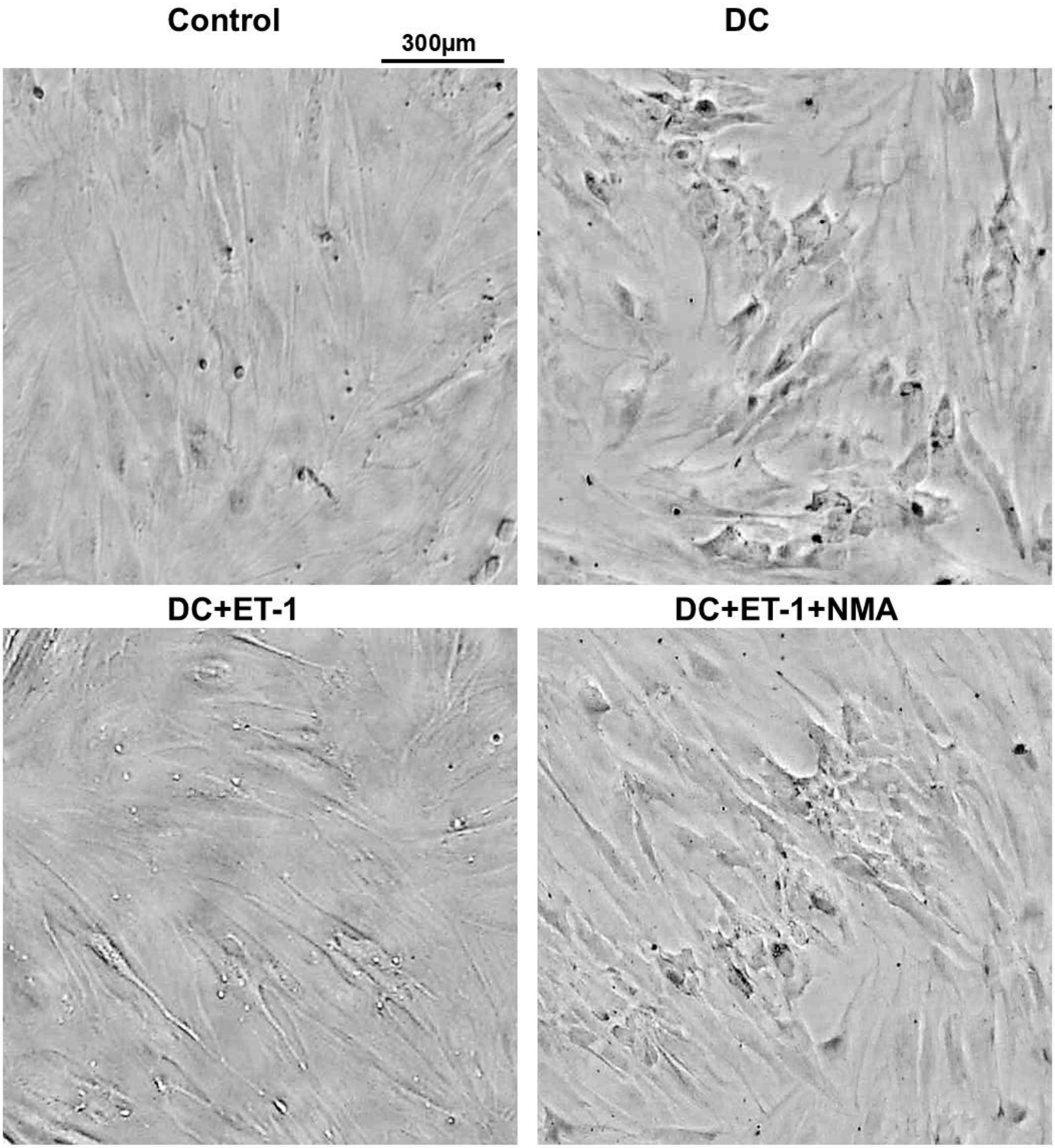

Supplemental Figure S5. The morphology of stromal cells six days after decidualization. Control (C) cells had a typically fibroblastic appearance. Cells with DC had characteristics of decidual cells: larger and multinucleated. Cells with DC and ET-1 exhibited more fibroblastic-like features. NAM restored the characteristics of decidual cells in cells with DC and ET-1.

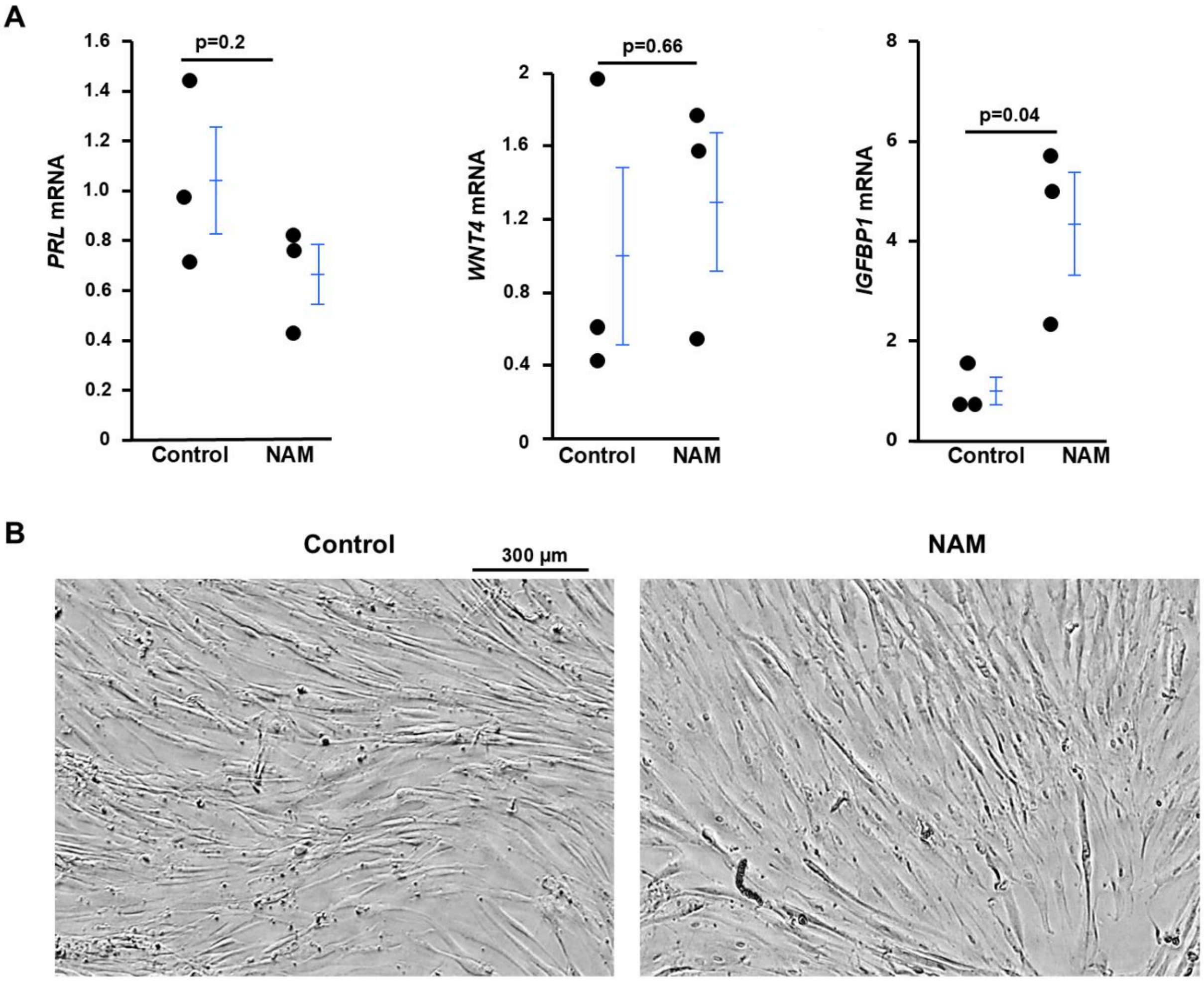

Supplemental Figure S6. Nicotinamide (NAM) has minimal effects on non-decidualized endometrial stromal cells. A. The expression of markers of decidualization. t-test. n=3. **B.** NAM did not chang the morphology of non-decidualized stromal cells. A high dose of NAM (10 mM) was applied.

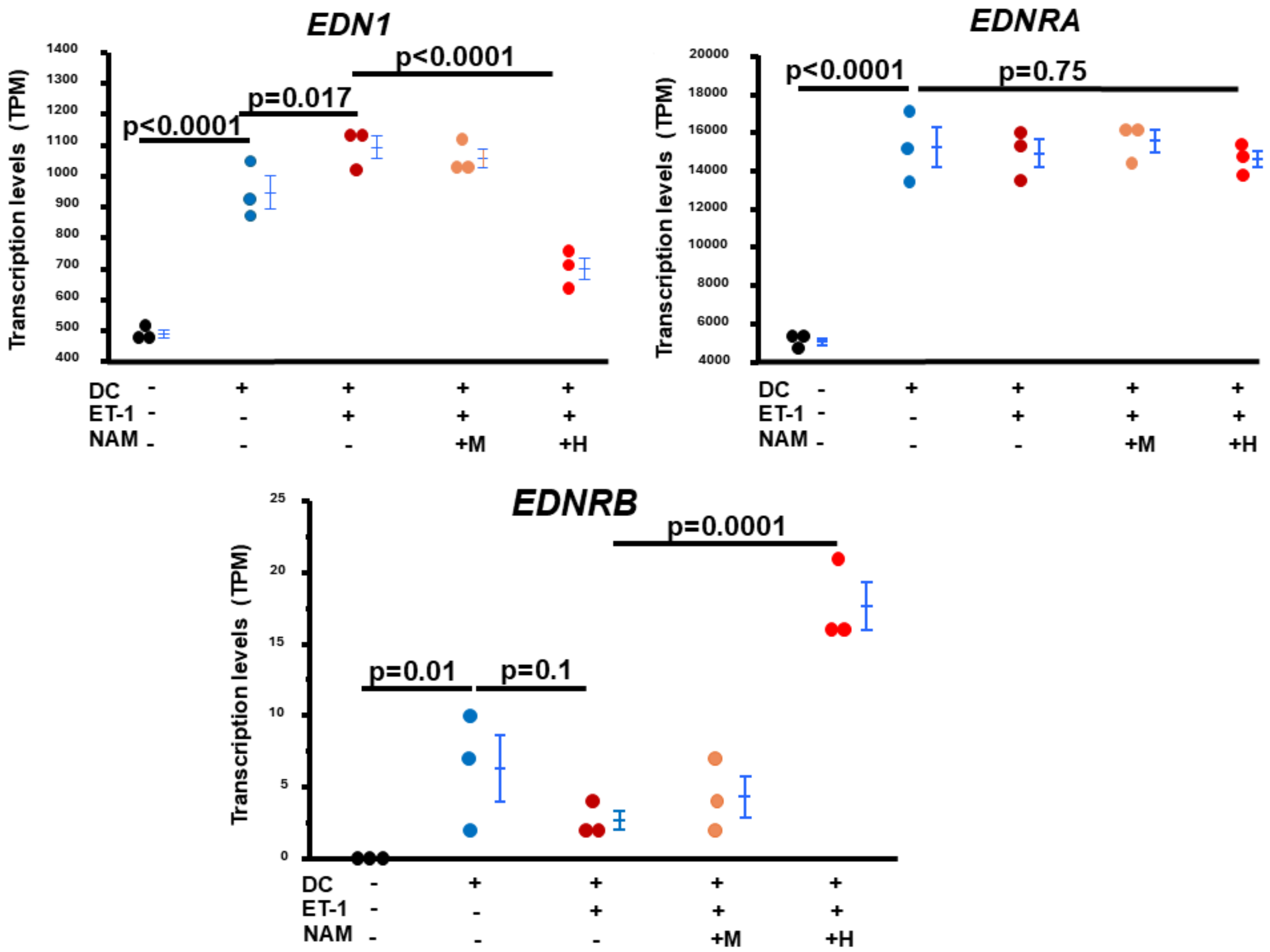

Supplemental Figure S7. Nicotinamide (NAM) executes distinct effects on the expression of *EDN1* and its two receptors. Transcriptional levles of *EDN1* (**A**), *EDNRA* (**B**), and *EDNRB* (**C**) were determined by RNA-seq analysis three days after a decidualization cocktail (DC) exposure with or without ET-1/NAM. M: medium dose of NAM (1 mM), H: high dose of NAM (10 mM). Student t-test. n=3.

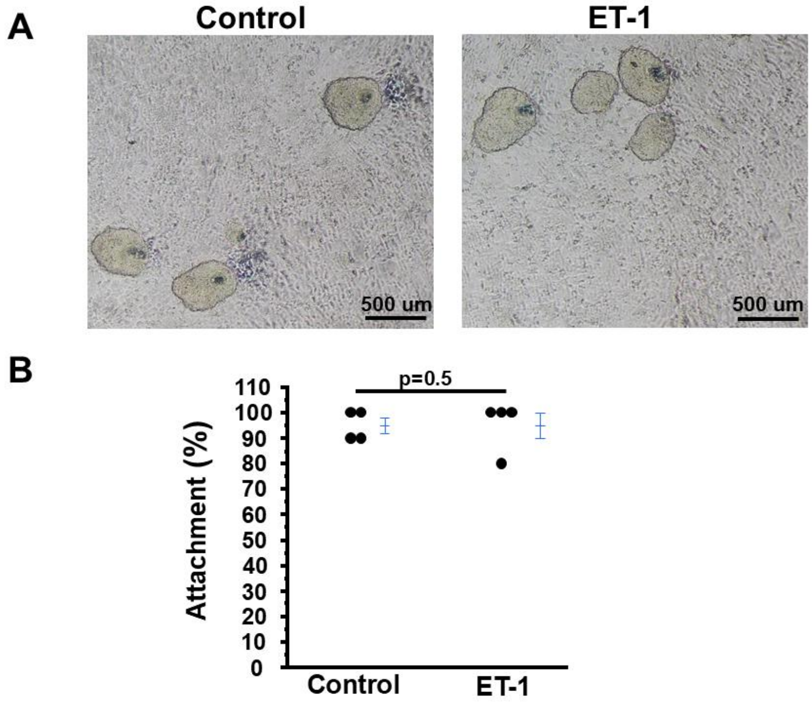

Supplemental Figure S8. ET-1 does not alter trophoblast spheroid attachment to Ishikawa cells (endometrial epithelial cells). The concentration of ET-1 applied was 0.5 μM. Two-tailed t test. n=4.
